# Improving cellular uptake of therapeutic entities through interaction with components of cell membrane

**DOI:** 10.1080/10717544.2019.1582730

**Published:** 2019-03-24

**Authors:** Renshuai Zhang, Xiaofei Qin, Fandong Kong, Pengwei Chen, Guojun Pan

**Affiliations:** a Key Laboratory of Flexible Electronics & Institute of Advanced Materials, Jiangsu National Synergetic Innovation Center for Advanced Materials (SICAM), Nanjing Tech University, Nanjing, P.R. China;; b Key Laboratory of Biology and Genetic Resources of Tropical Crops, Ministry of Agriculture, Institute of Tropical Bioscience and Biotechnology Chinese Academy of Tropical Agriculture Sciences, Haikou, P.R. China;; c School of Life Sciences, Taishan Medical University, Tai’an, P.R. China

**Keywords:** Cellular uptake, delivery systems, plasma membrane, pro-drugs, disulfide exchange

## Abstract

Efficient cellular delivery of biologically active molecules is one of the key factors that affect the discovery and development of novel drugs. The plasma membrane is the first barrier that prevents direct translocation of chemic entities, and thus obstructs their efficient intracellular delivery. Generally, hydrophilic small molecule drugs are poor permeability that reduce bioavailability and thus limit the clinic application. The cellular uptake of macromolecules and drug carriers is very inefficient without external assistance. Therefore, it is desirable to develop potent delivery systems for achieving effective intracellular delivery of chemic entities. Apart from of the types of delivery strategies, the composition of the cell membrane is critical for delivery efficiency due to the fact that cellular uptake is affected by the interaction between the chemical entity and the plasma membrane. In this review, we aimed to develop a profound understanding of the interactions between delivery systems and components of the plasma membrane. For the purpose, we attempt to present a broad overview of what delivery systems can be used to enhance the intracellular delivery of poorly permeable chemic entities, and how various delivery strategies are applied according to the components of plasma membrane.

## 1. Introduction

Cellular exchange of substances and signaling is the most important processes for biological activity, which is strictly regulated by plasma membrane (a thin layer, 4–10 nm). On the one hand, plasma membrane as the outer boundary can protect cells from surrounding harsh environment, ensure the relative stability of the intracellular environment and enable various biochemical reactions to run in an orderly manner. On the other hand, plasma membrane is also the first barrier for therapeutic agents enter cells, which limit the development of large of potential drugs. Generally, the inefficient cellular delivery is the key factor limited the utility of therapeutic agents, since most therapeutic entities are usually designed to modulate intracellular components. It seems no problems for small molecules to enter cells. Three ways for them to internalize into cells: simple diffusion, facilitated diffusion, and active transport. However, some hydrophilic small molecule drugs showed inefficient bioavailability due to poor membrane permeability. Therefore some of them are designed to be pro-drugs for enhancing the lipophilicity and thus membrane permeability (Rautio et al., 2008). Namely, the cellular uptake of small molecule drugs should not be ignored. When molecules are too large (e.g. proteins) to cross the plasma membrane, cells could capture these substances through endocytosis. Nevertheless, in most cases, the efficiency of cellular uptake is not enough for macromolecule drugs (e.g. proteins) to accomplish their biological functions. In order to increase their cellular uptake, extensive efforts have led to the development of effective delivery systems that invoke cell-penetrating peptides (CPPs), antibodies, dendrimers, functionalized polymers, liposomes, nanoparticles, and so on (Chou et al., 2011; Allen & Cullis, 2013; Pisa et al., 2015). In general, all these delivery strategies for small molecules and delivery systems for large size chemic entities make up a complex field of drug delivery. The uptake of chemic entities showed various delivery mechanisms, including clathrin- and caveolin-dependent endocytosis, thiol- and counterion-mediated cellular uptake, etc (Mosquera et al., 2018). Either way, the first step for any chemic entities into cells is to interact with cell membrane. Therefore, the review will focus on how the components of plasma membrane affect cellular uptake. In other words, how utilize different components of plasma membrane to promote cell uptake of chemic entities.

Three main components of plasma membrane are lipids, proteins, and saccharides. Lipids are the main skeleton of cell membrane, which constitutes the boundary between cell and its surroundings, and play active roles in regulating numerous processes in cell physiology (van Meer et al., 2008). Proteins are embedded in lipids in different ways, they regulate the exchange of substances between internal and external media and provide cellular signaling (Filomeni et al., 2003; Kagatani et al., 2010). Inside the cells, carbohydrates provide energy for cell activity. Meanwhile, cell surface is coated with a dense forest of polysaccharides conjugated to proteins and lipids. Great advances in glycomics, reveal the scope and scale of their functional roles, and their impact on human disease (Hart & Copeland, 2010). In this review, we will present how different chemic entities internalize into cells via interacting with various components of membrane, and give our personal view of how to utilize various components of cell membrane to enhance cellular uptake of chemic entities, including small molecules, macromolecules, and drug carriers.

## 2. Cellular uptake by interacting with lipid

**Figure 1.**
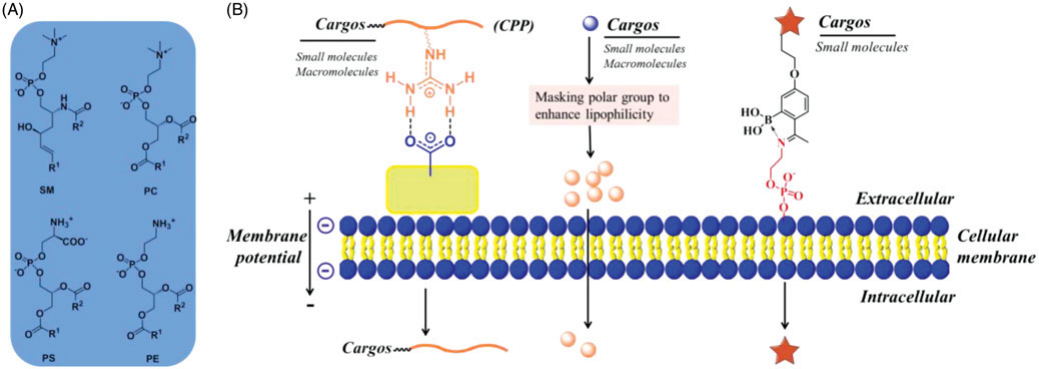
(A) Structures of major membrane lipids (sphingomyelin (SM), phosphatidylcholine (PC), phosphatidylethanolamine (PE) and phosphatidylserine (PS)). (B) A simplified representative illustration of uptake mechanisms through interaction with lipid.

Phospholipid bilayer is the basic scaffold of cell membrane, which consist of a hydrophobic and a hydrophilic portion (van Meer et al., 2008). The hydrophobic moieties propend to self-associate (entropically driven by water), and the hydrophilic moieties tend to interact with aqueous environments, thus spontaneously form phospholipid bilayer. Membrane lipids contain three major structural lipids, glycerophospholipids, sphingolipids, and cholesterol, respectively. Glycerophospholipids primarily consists of phosphatidylcholine (PC), phosphatidylethanolamine (PE) and phosphatidylserine (PS) (Figure 1(A)). Sphingomyelin (SM) is the major sphingolipid in mammalian cells. Cholesterol is the major non-polar lipids of cell membranes. Except for cholesterol, the hydrophobic portions are diacylglycerol and ceramide, which contains saturated or unsaturated fatty acyl chains of varying lengths. The three major hydrophobic portions are highly associated with the cell uptake of lipophilic small molecules by simple diffusion. Because soluble in lipid bilayer is a prerequisite for small molecules penetrate into cells by simple diffusion. The hydrophilic portion consists of phosphate and nucleophilic amine (in PS and PE)/quaternary amine (in PC and SM), the former can interact with guanidine to form a bidentate bond and the latter can be captured by 2-acetylphenylboronic acid to form an iminoboronate (Figure 1(B)). The cellular uptake of a large of cargoes (including small molecules, macromolecules, and drug carriers) may be enhanced by interacting with phospholipid bilayer described as above.

## 2.1 Improving uptake by interacting with hydrophilic portion of lipid bilayer

The hydrophilic portion of lipid bilayer contains phosphate groups and amines, which are the negatively charged polar headgroups of glycerophospholipids and sphingolipids. They can also interact with chemic entities and enhance their cellular uptake. Arginine-rich CPPs are the most successful example of enhancing cell uptake by the electrostatic interaction with negatively charged cell membrane (Rousselle et al., 2002). Generally, CPPs are short cationic peptides less than 30 residues, showing capability of traversing cell membranes without harming cellular integrity. Meanwhile, they have been successfully used to intracellular delivery of a wide range of biologically active molecules and drug carriers (e.g. nanoparticles). To date, the mechanisms for cellular internalization of CPPs have not been fully clarified. Nonetheless, it has become clear that the CPPs were adsorbed to negatively charged cell membrane prior to endocytosis. Generally, CPPs contain several cationic residues, such as Arg and Lys, which are widely recognized to play principal roles in the interaction with cell membrane. Most of Arg’s and Lys’s non-covalent bonding with anionic groups on cell surface comes from guanidinium and ammonium groups. The highly basic guanidinium and ammonium groups remain protonated under physiological pH conditions, and thus can function as hydrogen bond donors in CPP-lipid interactions. The H-bonding interactions of Arg guanidinium-phosphate and Lys ammonium-phosphate have been identified through solid-state NMR (Figure S2) (Su et al., 2009, 2010). Except for these interactions, Arg and Lys also form bidentate bonds with negatively charged sulfates and carboxylates which from glycosaminoglycan (GAG) of cell surface, the more details would be shown below (Section 8).

**Figure 2.**
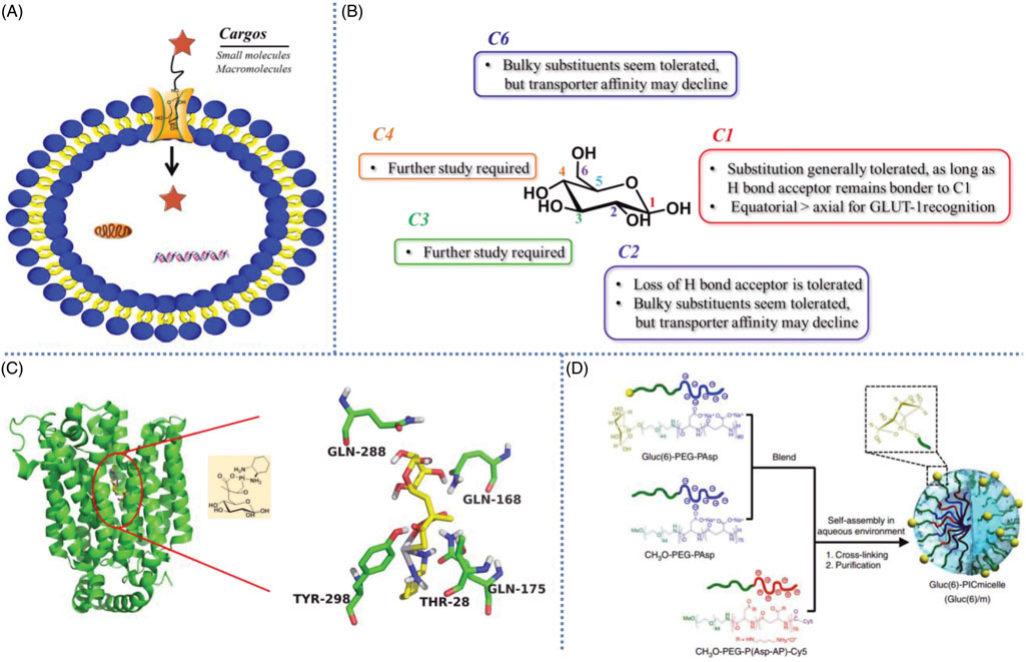
(A) GLUT mediated cellular uptake. (B) Structure-activity relationship of glucose as a substrate for the GLUT1 transporter. (C) The amino acid residues involving the hydrogen-bonding interactions present in the docking model of glucose-platinum conjugate into XylE (PDB 4GBZ). (D) Glucose was introduced onto nanocarriers to enhance retention. Reproduced with permission from ref (Calvaresiet al.,2013). Copyright 2013 Royal Society of Chemistry.

Direct guanidinylation of small molecular entities have been proved to effectively enhance cellular uptake. Luedtke et al*.,* revealed that guanidine modification to tobramycin and neomycin B, antibiotic natural products with poor cellular uptake, remarkably increase their uptake efficiency (Luedtke et al., 2003). The studies showed that the cellular uptake of guanidine-mediated tobramycin was ∼10-fold higher than natural tobramycin, and guanidinylated neomycin B also showed significantly enhanced cellular uptake (∼20-fold). Additionally, they shared similar uptake mechanism to that of CPPs.

For macromolecules and drug carriers, poor permeability limits their delivery to the intended targets and thus their bioavailability for the therapy. CPPs have been utilized to overcome those limitations through the enhancement of the attraction between macromolecules and negatively charged cell membrane. Many studies showed that CPPs could be conjugated to macromolecules, such as peptide, protein, and nucleic acid, for facilitating their transduction into cells (Futaki, 2002; Bechara & Sagan, 2013). Early in 1999, Schwarze et al., has reported that β-galactosidase (120 kDa) was delivered in its active form to all tissues, including the brain, through fusing the cargo to TAT (transactivator of transcription) peptide (Schwarze et al., 1999). The first example of CPP-mediated nanoparticle delivery was also described in 1999. Josephson et al., reported that TAT peptide modified iron oxide nanoparticle was internalized into cells over 100-fold more efficiently than non-modified nanoparticle (Josephson et al., 1999). Moreover, CPPs, as a non-viral vector, have been extensively used for the delivery of nucleic acids both *in vitro* and *in vivo* (Lehto et al., 2012). The studies of Torchilin et al. indicated that even relatively large drug carriers, such as 200-nm liposomes, can also be successfully delivered into cells by TAT peptide attached to the liposome surface (Torchilin et al., 2001). On the other hand, direct guanidinium modification has also been used for enhancing cellular uptake of peptide nucleic acids and DNA (Zhou et al., 2003; Ohmichi et al., 2005). Further, a series of guanidinium-decorated peptides, carbohydrates, oligocarbamates, and dendrimers have also been found to exhibit highly efficient cellular uptake similar to that of CPPs, and been used to deliver cargos as varied as small molecules, macromolecules, and carriers (Wender et al., 2002; Maiti et al., 2006; Huang et al., 2007).

## 2.2 Improving uptake by interacting with hydrophobic portion of lipid bilayer

Cholesterol, diacylglycerol, and ceramide are the main hydrophobic components of lipid bilayer. The cellular uptake of many chemic entities, especially small molecules, is closely related to the hydrophobicity of cell membranes. Small molecules can cross plasma membrane into cells by simple diffusion as they can be soluble in the hydrophobic region of phospholipid bilayer. Lipophilicity is one of the main parameters that determine cell uptake of small molecules. Generally, when small molecules cross lipid bilayer by simple diffusion, they firstly accumulate in the hydrophobic regions of lipid bilayer at high concentration through hydrophobic interaction. Thus, small molecules must have moderate lipophilicity in order to internalize into cells. On the other hand, some membrane anchoring moieties (e.g. cholesterol, squalene, and fatty acids) can interact with the hydrophobic tail regions of the lipid bilayers and promote the cellular internalization of chemic entities. In some cases, hydrophobicity and lipophilicity could be used interchangeably although they are not synonyms. Thus, some strategies (including pro-drug and anchoring moieties modification) improved cellular uptake by interacting with hydrophobic portion were displayed in this section, without discussing whether they increase hydrophobicity or lipophilicity.

Pro-drug strategy has been used to improve the cell uptake of small molecules through increasing lipophilicity. At present, about 10% of drugs approved worldwide are administered as pro-drugs (Hajnal et al., 2016). In most cases, increasing lipophilicity is one of the important purposes for using of pro-drugs. In many small molecule drugs, charged groups such as the carboxylic acids and phosphates exist as indispensable functional groups for their pharmacological activity. However, their presence reduces the lipophilicity, and thus prevents the passage of molecules through membranes by simple diffusion. Masking these charged groups with aliphatic alcohol via esterification reaction is the most widely used strategy to enhance the lipophilicity, and thus the passive membrane permeability (Rautio et al., 2008). Oseltamivir is the ester pro-drug of the antiviral molecule oseltamivir carboxylate. Previous study showed that the oral bioavailability of oseltamivir increased to 80% after ester modification, while that of oseltamivir carboxylate is less than 5% (Doucette & Aoki, 2001). Adefovir dipivoxil is an oral pro-drug of the nucleotide analog adefovir. The study proved that the oral bioavailability increased to 30–45%, after esterifying the phosphate group. More examples on ester pro-drugs that enhance oral absorption of predominantly poorly permeable and polar parent drugs can be seen in Beaumont et al.’s review (Beaumont et al., 2003).

Proteins are also promising therapeutic agents, however, their cellular uptake is very inefficient. Apart from the anionic glycocalyx, the hydrophobic lipid bilayer is one of the barriers that proteins must overcome for crossing plasma membrane (Palte & Raines, 2012). Interestingly, Mix et al. presented an esterification strategy to cytosolic delivery proteins (Figure S1) (Mix et al., 2017). In their studies, cloaking carboxyl groups of green fluorescent protein (GFP) with a hydrophobic moiety (2-diazo-2-(p-methylphenyl)-N, N-dimethylacetamide) could enable GFP to enter the cytosol of CHO-K1 cells. The studies of uptake mechanism indicated that cellular uptake does not rely on endocytosis. Taking the cloaking carboxyl groups increasing hydrophobicity of GFP into consideration, it can be expected that decorated-GFP appears to enter cells by crossing directly the plasma membrane, like a small-molecule pro-drug.

Effective delivery of therapeutic entities is critical in view of their clinical application. Some membrane anchoring moieties, such as cholesterol and alkyl chain, have become a subject of considerable interest to improve the safe delivery of oligonucleotides (Raouane et al., 2012; Chen et al., 2018). These membrane anchoring groups are usual hydrophobic components of cell membranes (e.g. cholesterol), or analogs of hydrophobic components (e.g. squalene and vitamin E). They could increase accumulation of chemical entities on cell surface through interacting with hydrophobic tail regions and thus promote cellular internalization. It has been reported that cholesterol-PEG could preferentially bind to cholesterol-rich hydrophobic regions of lipid bilayer through hydrophobic interaction (Sato et al., 2004). The study of Jürgen Soutschek et al., showed that cholesterol modified siRNAs can silence an endogenous gene encoding apolipoprotein B (ApoB) in HepG2 cells with no need of transfection reagents or electroporation. Further, systemic administration of cholesterol decorated ApoB-siRNA obviously reduced the level of ApoB mRNA in liver and jejunum after intravenous injection. This did not happen for unconjugated ApoB-siRNA in mice (Soutschek et al., 2004). Moreover, alkyl chain has also been used to modify siRNAs for efficient cellular delivery. The delivery efficiency mainly depended on the length of alkyl chain because that the hydrophobicity increases with the prolongation of alkyl chain and thus strengthen interaction with hydrophobic regions of the lipid bilayers (Petrova et al., 2012). It is worth noting that hydrophobic interaction may only be part of the driving force for cell uptake in siRNAs delivery, some trans-membrane proteins and lipoprotein receptors have also been shown to be associated with cholesterol- or alkyl chain-decorated siRNAs delivery (Wolfrum et al., 2007). Additionally, some nanoparticles were decorated with hydrophobic segments to promote their adhesion to the hydrophobic portion of lipid bilayer through hydrophobic interactions. Cholesterol modification can achieve the enhanced plasma membrane enrichment and endocytosis of fluorescent quantum dots (QDs) in cancer cells via lipid raft-dependent endocytosis (Wang et al., 2016). A similar example is cholesterol modification achieved efficient cytosolic delivery of nanomicelles depending on cholesterol moiety triggered the lipid raft mediated endocytosis (Jia et al., 2018).

## 3. Cellular uptake by interacting with proteins located on cell membrane

Proteins are the second major components of cell membranes and some of them can mediate cellular uptake termed also receptor-mediated uptake in general describing. In order to profound the understanding of key role of trans-membrane proteins in mediated drug delivery, they were divided into two categories, transporters and receptors in this review. For instance, some trans-membrane proteins are transporters that carry small molecules (e.g. glucose) into the cell. Some other proteins are known as receptors to mediate the cell signaling pathway for growth and proliferation. For transporters, they maintained the normal metabolism of cells via transferring necessary nutrients from the outside to the inside. Meanwhile, some transporters showed high affinity to ligand-drug conjugates and even ligand-drug carrier complexes. Thus, it provided an ideal opportunity for enhancing drug delivery and improving drug targeting. For receptors, they can be especially bound by natural ligands or monoclonal antibodies (mAbs), and thus mediated cellular signal or used in the treatment of disease. To date, lots of mAbs have been conjugated to small molecule drugs or drug carriers for drug delivery. Additionally, some transporters can also be targeted by mAbs, such as folate receptors (FR) which transport folate into cells, and meanwhile as antigens can specially bind anti-FR antibody. Importantly, covalent attachment of small molecule drugs or drug carriers to antibodies did not significantly influence their cell internalization, thus providing another delivery strategy utilizing interaction between antibody and membrane proteins.

## 3.1 Trans-membrane proteins as transporters mediated cellular uptake

Transporter-mediated transcytosis is using those agents that resemble the specific endogenous substrates to be taken up and deliver into cells. Transporters located on cell surface enable targeted delivery of chemotherapy drugs or other therapeutic agents containing specific substrates, which will significantly reduce the undesirable toxicity and increase the efficacy of treatment. Several transporters have been indicated as potential targets for the specific delivery of therapeutic agents, including transferrin receptor (TfR), folate receptor (FR), glucose transporters (GLUT), integrin receptor (IR), cell adhesion molecule (CAM) receptor, etc. They can serve as a compelling way to deliver many potent therapeutic agents, ranging from small drugs to large drug carriers.

### 3.1.1 Delivery via TfR

Transferrin receptor (TfR) is a type II trans-membrane glycoprotein, which can bind avidly to transferrin (Tf, a circulating iron binding protein produced by liver and brain membranes) to mediate cellular iron uptake via endocytosis pathway. The cellular uptake path way has been efficiently exploited for drugs delivery. Tf-conjugated drugs or drug-carriers have higher uptake and accumulation in overexpressed TfR cells, as shown in Figure S3. Li et al., reported that transferrin promoted the internalization of transferrin-coupled liposomes (Li et al., 2009). Further, MBP-426 (Mebiopharm), a liposome loaded with oxaliplatin (L-OHP), was conjugated to transferrin (Tf) for tumor targeting and currently undergoing phase II trials (van der Meel et al., 2013). Sahoo et al. disclosed the intracellular uptake of transferrin-conjugated nanoparticles (drug carriers) was about three times higher than that of unconjugated nanoparticles in PC3 cells (Sahoo et al., 2004). Choudhury et al. summarized transferrin receptors-targeting nano-carriers for efficient targeted delivery and transcytosis of drugs into the brain tumors by conjugating Tf ligand to nano-carriers (Choudhury et al., 2018). What needed to emphasize is that, Tf is a single chain glycoproteins containing 700 amino acid residues with the molecular mass of 80 kDa. Immunogenicity may be the biggest disadvantage for macromolecule ligand (e.g. Tf, lectin) mediated cellular uptake and targeting. On the contrary, low molecular weight ligands are presumably non-immunogenic and can be administered repeatedly, such as the ligands of GLUT and FR. They have been exploited to enhance cellular uptake and caner targeting. Importantly, some agents have been in clinical trial and showed significant potential for future clinical application.

**Figure 3.**
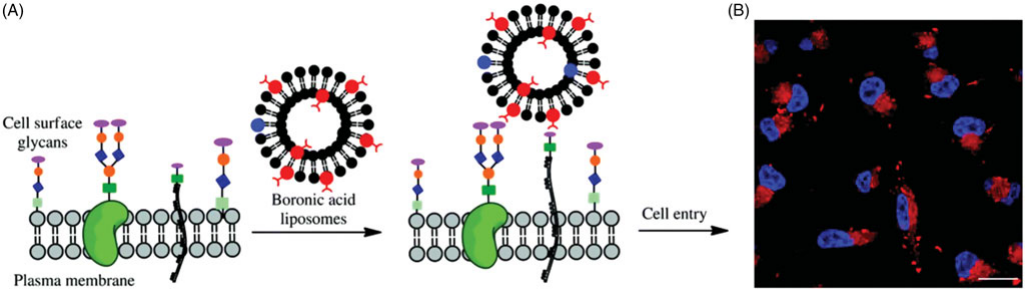
(A) Cartoon depicting liposome cell entry driven by binding interactions with cell surface carbohydrates. (B) Fluorescence image of cells treated with boronic acid modified liposomes. Reproduced with permission from ref (Zhang et al., 2018). Copyright Royal Society of Chemistry.

### 3.1.2 Delivery via GLUT

Glucose transporters (GLUT), including GLUT1, GLUT2, GLUT3, GLUT12, and SGLT1/2 and so on, could mediate saccharides or saccharide-conjugates’ cellular uptake (Figure 2(A)) (Calvo et al., 2010; Szablewski, 2013). Thus, GLUT-based (especially GLUT1) delivery strategy has garnered a great deal of interest and has grown markedly in recent years. For example, glycoconjugation have become an appealing strategy for targeted delivery of anticancer drugs due to the overexpress of GLUT1 in cancer compared to normal tissues. In order to utilize the GLUT1-based delivery strategy to enhance cellular uptake or to selectively target cancer cells, aglycone should not hinder the interaction between glucose and GLUT1. Calvaresi et al. summarized structure-activity relationship of D-glucose as a substrate for the GLUT1 transporter (Figure 2(B)) (Calvaresi & Hergenrother, 2013). Substitutions at C1, C2, and C6 have been most explored to date, some generally substitution seems to be tolerated, while substitutions at C3 and C4 require further study. Some substitutions at C1, C2, and C6 can be made with the resulting conjugates retaining affinity for GLUT1. Especially the C6 position of D-glucose, can tolerate various functional groups while retaining affinity for, and internalization by, GLUT1. Furthermore, C6-glucose conjugates of 4-nitrobenzofurazan, ketoprofen, and indomethacin were reported to bind GLUT1 with even higher affinity than unmodified D-glucose (Speizer et al., 1985; Barros et al., 2009; Gynther et al., 2009). It was worth noting that the C1, C2, and C6 positions of D-glucose showed higher reactivity than that of C3 and C4, therefore, the conjugates with C1, C2, and C6 substitutions were more easily obtained, that maybe one of reasons why C1, C2, and C6 substitutions have been widely studied. Although lots of conjugates with C3 and C4 substitutions have been synthesized, to our knowledge, there were no any reports about the affinity between C3- or C4-glucose-conjugated compounds and GLUT1. Hopefully, Deng et al. reported firstly the crystal structure of human GLUT1 in 2014, which make it possible that utilizing computer simulation to predict the interaction between GLUT1 and substrates, especially C3- or C4- glucose related interaction (Deng et al., 2014). This exciting study would greatly promote the development of GLUT1-based active molecules and drug delivery platform.

In the field of small molecule drug delivery assisted by GLUT, glufosfamide was the first sugar conjugate to be explicitly designed and evaluated as a cancer-targeting cytotoxic small molecule, which was initially reported by Wiessler and colleagues in 1995 (Pohl et al., 1995). The results showed that anticancer potency of glufosfamide was markedly reduced upon co-treatment with 0.1 mM of the GLUT-1 transporter inhibitors, suggesting that the cellular uptake of glufosfamide was at least partially GLUT1-mediated. Further, recent study revealed that platinum-based anticancer drugs conjugated with glucose were transported by GLUT1 and showed selectivity against cancer cell (Figure 2(C)) (Patra et al., 2016). They preferentially accumulated in and annihilated cancer, compared to normal epithelial, cells *in vitro*. The cellular uptake of glucose-platinum conjugate was reduced to 50%, when the ovarian cancer cell line A2780 (high level of GLUT1 expression) were treated with GLUT1 inhibitor 4, 6-O-ethylidene-α-D-glucose (EDG), indicating that GLUT1 was involved in the uptake of glucose-platinum conjugate. The similar results were also observed in our previous studies, which showed that GLUT1 played an important role in cellular uptake of ribose-modified antitumor compounds in A549 cell line (Zhang et al., 2017). Above 30% reduction in cellular uptake was measured in the presence of 50 mM EDG. Additionally, the results suggested that other saccharide, beside D-glucose, could also be substrates for GLUT1, and thus can be considered candidates for a GLUT1-based delivery strategy. Actually, some other saccharides, e.g. D-mannose, D-galactose and D-xylose, were also transported into cells in a GLUT1-mediated fashion (Melisi et al., 2011).

Furthermore, GLUT-mediated transportation also showed potential application in delivery of drug carriers. Uchida et al. prepared a self-assembled glucose-integrated supramolecular nano-carrier, which could cross blood-brain barrier (BBB) and accumulate in brain due to the rapid glycemic increase after fasting and by the putative phenomenon of the highly expressed GLUT1 in brain capillary endothelial cells (BCECs) migrating from the luminal to the abluminal plasma membrane (Figure 2(D)) (Uchida et al., 2011). The surface of the polymeric micelle nano-carrier was decorated with multiple glucose molecules with a controlled density. Thus, the strong retention of nano-carriers was achieved by multivalent interaction between a nano-carrier and multiple GLUT1 molecules on BCECs. This multi-molecules decorated strategy was generally used in macromolecule drug delivery system. Qin et al. reported a brain targeting drug liposome carrier with glucose-cholesterol derivative as ligand (Qin et al., 2010). The novel liposome could overcome the ineffective delivery of normal drug formulations to brain through interaction with GLUTs on the BBB. The glucose-decorated liposome showed higher brain concentration and AUC_0–t_ compared to control liposome after i.v. administration. Thus, it’s a promising strategy to take advantage of GLUT1-mediated cellular uptake through conjugate many saccharides to chemical entities needed to be delivery.

### 3.1.3 Delivery via FR

Folate receptors (FR-α and FR-β) are cysteine-rich cell-surface glycoproteins, which bind folate and folate conjugates with high affinity (K_d_ ∼10^−9 ^M) to mediate their cellular uptake (Chen et al., 2013). Similar to GLUT1 overexpression in cancer, folate receptor α (FR-α) has been demonstrated to be overexpressed in an estimated 40% of human cancers, which make it possible to utilize folate receptor to enhance cellular uptake and improve cancer selectivity (Low & Kularatne, 2009). A wide range of small molecular chemotherapeutics and drug carriers (e.g. nanoparticles) have been conjugated to folate and thus evaluated for FR mediated cellular uptake and FR targeting. In all tissues where FR is expressed, folate conjugates (including small molecules and drug carriers) were internalized via receptor-mediated endocytosis, although the endocytosis of small molecules and macromolecule folate conjugates displayed different itinerary.

In the endocytosis process of folate decorated small molecular conjugates, the folate ligand firstly binds to FR and thus induces cell membrane invaginate to form an early endosome. Subsequently, the early endosome was transported immediately to a recycling center nearby the cell nucleus, where the conjugates were separated into distinct vesicles, whereas the FR was moved to another endosome that recycled back to the cell membrane to start a second round of transport by binding with new folate-targeted conjugates (Chatterjee et al., 2001; Paulos et al., 2004). Because early endosomes contained considerable reducing power, allowing for the rapid release of drugs linked to folate via a disulfide bond, hence, using disulfide to construct folate decorated small molecular conjugates was very promising and viable strategy (Yang et al., 2006). Actually, some clinically tested folate conjugated drugs were indeed designed based on disulfide bond linker. EC-145, a folate conjugated drug of desacetylvinblastine hydrazide, performed well in preclinical and has been in phase III trials for the treatment of ovarian cancer in the US (Reddy et al., 2007). A disulfide bond was used to connect the folate spacer moiety and the desacetylvinblastine hydrazide, which could be cleaved by endosomal cathepsins and released the cytotoxin intracellularly. Importantly, the present of folate moiety enhance uptake of cancer cell and decreased toxicity at doses where evidence of anti-tumor activity was observed. Additionally, several small molecular imaging agents were also attached to folate for targeted delivery to FR-overexpress cells. They have been summarized in other review articles (Zhao et al., 2008). Although folate decorated strategy is useful for FR-targeted drug delivery, the limitations should be pay attention. The membrane permeable therapeutic cargo can be attached and transported into cytoplasm, however, non- membrane permeable molecules were not desirable since they can’t escape from endosomes containing no pores or channels. Thus, the FR-mediated cellular uptake only makes benefit to those membrane permeable drugs.

In addition to small molecules, marcomolecules and drug carriers have also been conjugated to folate. Leamon et al. firstly reported folate decorated proteins could be deliverrd into KB cells via endocytosis pathway in 1991 (Leamon & Low, 1991). Li et al. reported the uptake of folate modified oligodeoxyribonucleotides (ODNs) was increased about 8-fold compared with controls in FD2008 cells overexpressed folate receptors (Li et al., 1998). The increase could be blocked by adding an excess amount of folic acid. In contrast, the uptake did not increase in CHO cells that lack the expression of FR. The results showed the biological activity of ODNs was significantly increased with the enhancement of uptake on FD2008 cells, indicating that the uptake of folate decorated ODN conjugate was mediated by FR. After that, several protein toxins, such as momordin, pseudomonas exotoxin, and gelonin have been conjugated to folate and shown selective cytotoxicity toward FR-overexpressed cells (Leamon & Low, 1993). As for the endocytosis of folate-decorated drug carriers, which could be transported quickly into lysosomes after FR binding, where both FR and any digestible cargoes were degraded by hydrolases in lysosome (Yang et al., 2007). The average pH of lysosome (about 5.0) was lower than cytoplasm, which provided a uniquely method for pH-triggered drug release through acid labile linkers coupling carriers to folate (Lee et al., 1996). Folate-decorated drug carriers showed high affinity multivalent interaction to the FRs because multiple folates were typically conjugated to each particle. However, they also displayed more rapid clearance via the liver the spleen than unmodified carriers due to their greater affinity to reticuloendothelial system cells and some level of expression of FR-β on regular macrophages (Gabizon et al., 2003). The advantages of using FR-targeted drug carriers warrant further investigation.

## 3.2 Trans-membrane proteins as receptors mediated cellular uptake

Receptor-directed drug targeting is an active area of research and has the potential to provide novel and powerful treatments for diseases. Meanwhile, much effort has been made to utilize receptor-mediated transcytosis/endocytosis (RMT) to enhance cellular uptake in drug delivery field. RMT is the binding of targeting ligands (e.g. peptides, antibodies) to the receptors (protein biomarkers/antigens) expressed on the surface of the cell. Unlike transfection reagents, as well as, cell penetrating peptides lacking cell-type specificity, receptor-mediated delivery provides a better opportunity for optimal targeting of therapeutic agents, which could deliver a wide variety of therapeutic entities without compromising the integrity of the cell membrane.

The epidermal growth factor receptor (EGFR) is a large trans-membrane glycoprotein (180 Kda). Althoug EGF is the most commonly endogenous ligand for EGFR, and has been used to decorate drug carriers for increasing cellular uptake and drug accumulation in cancerous cells (Tseng et al., 2007). Monoclonal antibody (mAb) was still the most ideal strategy to target EGFR in drug delivery field. Anti-EGFR antibody, targeting the extracellular domain of EGFR, has been used as targeting agents to selectively deliver chemotherapeutics to cancerous cells. Elegant work conducted by Acharya et al., disclosed that cellular uptake of EGFR antibody conjugated nanoparticles (dye loaded) was nearly 13 times more than unconjugated nanoparticles, and 50 times more than free dye (Acharya et al., 2009). The higher cellular uptake was attributed to greater intracellular delivery by receptor mediated endocytosis. On the other hand, the studies of Bhattacharyya et al. found that the nano-conjugation cannot be construed as an innocuous reaction involved in attaching cetuximab (anti-EGFR antibody) to the Au NPs, instead it may distinctly alter the cellular processes at the molecular level, at least in antibody induced receptor endocytosis (Bhattacharyya et al., 2010). This information was critical for successful design of antibody-NP complexes drug delivery systems for future clinical translation. Mamont et al. reported the immunoliposomes (Ils) loaded with DOX targeted EGFR overexpressing tumors via coupling of Fab fragments of the anti-EGFR mAb cetuximab (Mamot et al., 2003). *In vitro* studies showed ∼30-fold more EGFR-positive cell internalization of anti-EGFR Ils compared to non-targeted liposomes. Moreover, studies in rats showed conjugation of Fab did not alter liposomal stability or circulation time. The anti-EGFR Ils-DOX has been recommended for phase II trials in 2012 (Mamot et al., 2012).

Epithelial cell adhesion molecule (Ep-CAM) is an epithelial cell mono-subunit trans-membrane glycoprotein (Went et al., 2006). Upon binding to Ep-CAM specific ligands, Ep-CAM receptor is rapidly internalized and thus ideally suited for drug delivery. Hussain et al. prepared Ep-CAM targeted immune-liposomes (SIL25) through covalently linked humanized single-chain Fv antibody fragment 4D5MOCB to the exterior of coated cationic liposomes, which showed specific binding to Ep-CAM-overexpressing tumor cells, with a 10–20-fold increase in binding compared with non-targeted control liposomes by receptor-mediated endocytosis (Hussain et al., 2006).

Prostate specific membrane antigen (PSMA), a type 2 integral membrane glycoprotein, overexpressed on prostate cancer epithelial cells and has been used to facilitate drug delivery. Farokhzad et al. prepared docetaxel-encapsulated NPs and surface functionalized with the A10 2′-fluoropyrimidine RNA aptamer (Apt) that recognize the extracellular domain of the PSMA of prostate cancer cells (Farokhzad et al., 2006). The Apt functionalized NPs bind to the PSMA protein and get taken up by these cells, resulting in significantly enhanced *in vitro* cellular toxicity as compared with NPs lacked the PSMA aptamer. Cheng et al. developed surface functionalized with A10 RNA Apt NPs, which showed significantly enhanced delivery efficiency compared with equivalent NPs lacking of the A10-PSMA Apt (a 3.77-fold increase at 24 h) in studies of mice (Cheng et al., 2007). Furthermore, Apt-decorated NPs showed remarkable antitumor efficacy *in vivo* after a single intratumoral administration. BIND-014 is a polymeric nanoparticle which target PSMA expressing cells using small-molecule as targeting ligand. In preclinical studies, optimized BIND-014 treatment caused significant tumor growth inhibition in a mouse compared to ligand-lacking controls. In contrast, no difference in anti-tumor effect was observed in PSMA- negative xenograft models. A phase II clinical trial of BIND-014 suggested that treatment with BIND-014 was active and well tolerated in patients with chemotherapy-naive metastatic castration-resistant prostate cancer (Autio et al., 2018).

Monoclonal antibody (mAb) binding TfR is another perfect way to exploit the TfR for drug delivery systems. The whole antibody or even single chain antibody fragments specific for the extracellular domain of the TfR can be used for drug delivery (Daniels et al., 2012). OX26, a mAb targeting TfR, was coupled to vasoactive intestinal polypeptide analogs by means of the avidin-biotin system to increase uptake of the peptide in the brain (Bickel et al., 1993). Niewoehner et al. developed a brain shuttle construct for treatment of Alzheimer’s disease through fusing the Fab fragment of an anti-TfR monoclonal antibody to the Fc region at the C-terminal end of the heavy chain of an anti-Aβ mAb (mAb31) (Niewoehner et al., 2014). The mAb31 has been shown to specifically bind with high affinity to β-amyloid plaques. The present of Fab (fragment of anti-TfR mAb) increased β-amyloid target engagement in a mouse model of Alzheimer’s disease by 55-fold compared to the free antibody. Up to date, the greatest progress has been made in the development of SGT-53, which was a cationic lipid and has progressed into clinical trials. SGT-53 was targeted to the TfR by a single-chain antibody fragment (scFv) to achieve successfully intracellular delivery of the plasmid DNA.(Senzer et al., 2013) It is noteworthy that the scFv has a smaller size than the Tf molecule and it allows large scale recombinant production and stricter quality control.

Conjugation to anti-FR antibody is another strategy for enhancement of drug delivery, which was mediated by FR as antigen. However, based on existing literatures, a vast majority of FR targeting delivery strategies utilize folate as the targeting ligands, that may be attributed to the limited availability of anti-FR antibody (Elgqvist et al., 2005). In this case, FR as folate transporter mediated folate conjugates cellular uptake, which has been discussed in above part.

Large protein ligands (e.g. antibodies) can be difficult to isolate or modify in a specific manner. In contrast, small molecule ligands are easily chemically synthesized, allowing the incorporation of various reactive groups to facilitate coupling to myriad biomolecular cargoes. Anthony A. Kossiakoff et al. presented a protein cargoes delivery method based on a variant of substance P (SPv), an 11 amino acid neuropeptide that is rapidly internalized through specific interaction with the neurokinin-1 receptor (NK1R) (Rizk et al., 2009). NK1R is a member of the tachykinin G-coupled receptor family, which highly overexpressed in brain tumor cells (Palma & Maggi, 2000). Because only the C-terminal portion of SPv is required for binding to the receptor, a wide variety of cargoes can be linked to the N terminus. In cargoes delivery studies, the novel strategy could specific deliver SPv-decorated proteins to NK1R-expressing cells via the interaction between SPv and NK1R, while internalization is undetectable in normal human astrocytes (NHA) without NK1R expression. It is important to note that, in many ligand-receptor delivery systems, the cargo appears to remain trapped in the endosome and thus might be biologically inactive, but the SPv-NK1R delivery system appear to readily escape the endosome without loss of function.

Integrins are a large family of heterodimeric trans-membrane proteins, which has been investigated intensively for drug delivery of various chemotherapeutic agents in the last decade. Integrin αvβ3, an internalization receptor for a number of viruses, has attracted the most attention in the field of drug delivery. The Arg-Gly-Asp (RGD) peptide was a high affinity ligand to αvβ3 integrin, has showed potential application in delivery systems (Schiffelers et al., 2003). Schiffelers et al. prepared a αvβ3-targeted self-assembling nanoparticle with RGD peptide ligands for overcoming the poor intracellular uptake of siRNA therapeutics (Schiffelers et al., 2004). FACS cell uptake quantification analysis indicated that the RGD modified NP showed 6- to 8-fold higher cellular uptake than unmodified NP. The uptake was inhibited by the addition of free RGD peptide via competitive bonding. These results suggested that the binding of RGD-NP complex to cells was mediated by the RGD peptide targeting ligand. Yu et al. also reported that RGD peptide can improve the nanoparticles’ cellular uptake in recently studies (Yu et al., 2015).

## 4. Cellular uptake by interacting with saccharides located on cell membrane

Saccharide is a complex and fascinating class of biomolecules, which play a key role in the activities of life (e.g. cell differentiation, cell-cell interactions) (Claes et al., 2013). In general, virtually all cell membranes contain proteoglycans and/or lipid glycans (Vandenburg et al., 2000). However, the role of saccharide on cell membrane seems to be highly variable and unpredictable. A given glycan on cell membrane can have different roles in different tissues or at different times in development (organism-intrinsic functions) or in different environmental contexts (organism-extrinsic functions). However, it’s clear that targeting synthetic biomolecules to saccharide on membrane could enhance their cellular delivery. Saccharides coated on cell membrane have showed potent in facilitating delivery of chemic entities.

Lectins are glycoproteins possessing at least one non-catalytic domain, which bind reversibly to specific mono- or oligosaccharides of the cell membrane (Peumans & Van Damme, 1995). Typically involving a high number of binding sites and determined by a specific sugar code, lectin binding is usually rapid and strong. Based on the outstanding binding properties, lectin-mediated drug delivery may become a promising strategy to improve the efficacy of poorly permeable drugs. Gabor et al. found that the dietary lectin wheat germ agglutinin can facilitate binding and uptake of protein drugs due to its cytoadhesive and cytoinvasive properties (Gabor et al., 2002). However, the potential disadvantage of natural lectins is of large size that results in immunogenicity and toxicity when they were used as drug carriers (Lehr & Gabor, 2004). One of the probable methods to overcome these problems is to design smaller peptides or even organic small molecules which can mimic the function of lectins. Several small size lectins (peptides) have been found, and shown better performance than natural lectins (Lü et al., 1999; Wang et al., 2003; Li et al., 2008). For example, odorranalectin, a lectin-like peptide from skin secretions of *Odorrana grahami*, showing extremely low toxicity and immunogenicity in mice (Li et al., 2008). However, the study of utilizing lectins as drug carriers was very little in recent 10 years compared to CPP and antibody delivery systems which have been discussed in above part. More efforts need to be put into the design field of lectins mimic before the ideal mimics were available.

As is mentioned above in Section 2, CPP has been used to overcome the obstacle of cellular uptake through enhancing the attraction between the chemotherapeutic agents and the anionic cell surface. Along with the lipids, proteoglycans are the other main component in the cell membrane which CPPs can interact with. Although the mechanisms of CPPs cell entry are still obscure, H-bonding between the guanidium groups of CPPs and the sulfates and/or carboxylates on cell surface GAG was considered to facilitate the internalization of CPPs complexes (Tyagi et al., 2001; Pujals et al., 2006; Poon & Gariépy, 2007). Actually, there is large abundance of negatively charged glycosaminoglycan (GAG) polysaccharides on cell surface, such as chondroitin sulfate (CS), dermatan sulfate (DS) and heparin sulfate (HS), which can also adsorb positively charged CPPs via electrostatic interactions. Console et al. reported the uptake of TAT, a widely used CPP, was greatly impaired in mutant CHO cells lacking HS and inhibited by both heparin and dextran sulfate, implying that the key roles of negatively GAG to enhance CPP-mediated cellular uptake (Console et al., 2003). Meanwhile, guanidinoglycoside, a guanidinium-rich molecular transporter, has been shown to facilitate the intracellular delivery of a diverse range of biologically relevant cargoes. Interestingly, their cellular uptake at nanomolar concentrations is exclusively HS-dependent, that is different from CPPs. The studies of Dix et al. showed that the uptake of guanidinoglycosides was negligible in mutant CHO cells lacking HS compared with that in wild type CHO cells (Dix et al., 2010). Furthermore, the uptake was diminished with the decrease of number of guanidinium groups. These findings showed that the key roles of negatively charged GAG in the cellular uptake of guanidinium-rich transporters.

Boronic acids were kinds of organic small molecules which can bind to saccharides. Their exceptional sugar-binding properties aroused the great interest of scientists. They could react spontaneously and reversibly with 1, 2- and 1, 3-diols of saccharides to form cyclic boronic esters, and the reaction has shown potential ability in chemical biology and pharmacology (Bosch et al., 2004; Peters, 2014). For instance, Phenylboronic acid (PBA) could react with 1, 2-cis-diols of glucose in blood to reversibly form the complex. Thus phenylboronic acid has been widely studied and used to construct glucose-responsive polymeric nanoparticles for insulin delivery in the treatment of diabetes. This smart delivery system can deliver and release insulin in a self-regulated way in response to the fluctuation of blood glucose concentration (Ma & Shi, 2014). In this review, we focus on the interaction between boronic acids and saccharide located on the surface of mammalian cells. This interaction has been used for diabetes and cancer diagnosis. For example, sialic acid (SA) is one of the biomarkers of diabetes, which could detect through the reversible and covalent interaction between PBA and SA at cell membrane (Otsuka et al., 2003; Matsumoto et al., 2009). Yang et al. designed several fluorescent sensors based on diboronic acid modification, which can fluorescently label cell through the specific recognition of cell-surface fucosylated carbohydrate. These fluorescent sensors could potentially be used for cancer diagnostic (Yang et al., 2004). These exceptional sugar-binding properties of boronic acid at physiological conditions indicate that saccharides at cell membrane may facilitate the cellar uptake of boronic acid modified biomolecules. Boronic acid modified cargoes were expected to bind to cell surface saccharides and enhance proximity to cell membranes, thereby boosting cell entry pathways such as membrane fusion or pinocytosis.

PBA has been used to enhance gene transfection capability of nonviral gene vectors (Peng et al., 2010). PBA-modified polyethylenimine (PEI) greatly enhanced gene transfection efficiency by two- to three-fold compared with unmodified PEI. Meanwhile, the uptake of PBA-modified PEI in SA-expressing HepG2 cells was much higher than that in non-SA-expressing COS-7 cells. These results suggested that the PBA-saccharide interaction on cell surface facilitated cell uptake of vector/DNA polyplexes. PBA has also been used to enhance liposomes delivery through the interaction with cell membrane saccharides (Vandenburg et al., 2000). Zhang et al. reported boronic acid decorated liposomes as a means for enhancing both cell infiltration and content delivery based on saccharides binding interactions (Figure 3) (Zhang et al., 2018). PBA modified liposome showed dramatic enhancement of delivery efficiency compared with control liposomes, which suggested that boronic acid is effective for driving liposomal cell entry. The PBA-conjugated chitosan nanoparticles were more easily internalized into cancer cells compared to non-decorated chitosan nanoparticles due to the interaction between over-expressed SA residues in cancer cells and PBA groups, thus more drugs could be delivered into cells (Wang et al., 2016). Deshayes et al. found that PBA-installed micelles showed much higher and faster cellular uptakes than micelles without PBA groups due to PBA’s specific target for SA groups overexpressed on tumor cells (Figure S4) (Deshayes et al., 2013). The antitumor effect of PBA-functionalized micelles was also enhanced due to the increased cellular uptake. 2-Hydroxymethylphenylboronic acid (benzoxaborole), combined structural features of boronic acids and cyclic boronic esters, has a greater affinity than PBA for saccharides (Dowlut & Hall 2006). Ellis and Andersen et al. reported that the benzoxaborole not only facilitated cellular uptake of proteins (e.g. GFP and RNase A) but also enhances their delivery to the cytosol through interaction with fructose, which is abundant in the glycocalyx on cell membrane (Ellis et al., 2012; Andersen et al., 2016).

**Figure 4.**
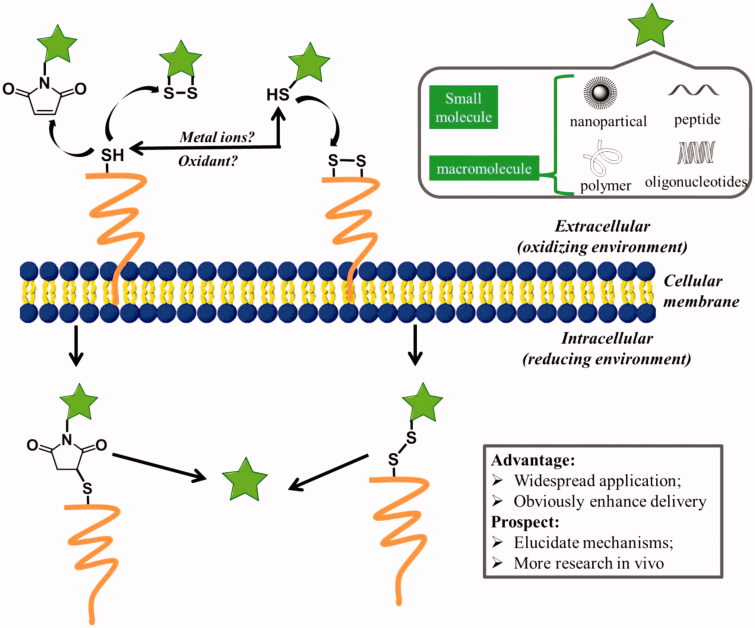
Possible mechanisms for cellular uptake of thiol-reactive groups (including maleimide moiety, disulfide bonds, and free thiols) modified biomolecules (pentacle) upon interaction with exofacial thiols. After these biomolecules were located on the cell surface, they were further internalized to free cargoes through cleavage.

Although boronic acid first gained attention as sugar-binding agents, the major development was connected with their biological action (Gupta & Simpson, 2015). The studies involving incorporation of boronic acid into materials for cargoes delivery was very little. However, the safety and effectiveness of boronic acid-decorated nanoparticles have been demonstrated in mice. Jiang et al. reported that PBA-decorated chitosan nanoparticles could penetrate deeper and accumulate more in tumor area than non-decorated ones. Furthermore, PBA-decorated chitosan nanoparticles showed superior efficacy in restricting tumor growth and prolonging the survival time of tumor-bearing mice than free drug and drug-loaded chitosan nanoparticles (Wang et al., 2016). Also, boronic acid-rich protein nanoparticles composed of bovine serum albumin (BSA) and poly(N-3-acrylamidophenylboronic acid) showed dominantly liver-targeting and significant washout resistant ability compared to those boronic acid-absent nanoparticles *in vivo* study, which was attributed to the interaction between SA residues in the liver and boronic acid groups of the nanoparticles (Wang et al., 2013). Additionally, there was no hepatic and cardiac toxicities *in vivo* antitumor examination in orthotopic liver cancer model. Deshayes et al. reported that the modification of PBA could maintain micelles’ accumulation level in the tumor even after 48 h injection due to the interaction between PBA and SA groups on the surface of cancer cells, whereas the amount of micelles without PBA decreased obviously (Figure S4) (Deshayes et al., 2013). The extension of retention time increased the anticancer efficiency and showed no side effects.

In summary, boronic acid is the widely studied molecules that interact with saccharide on cell membrane. With the help of saccharide on cell membrane, boronic acid-decorated carriers showed enhanced cellular uptake, and improved drug accumulation and retention in tumor. Accordingly, the treatment efficiency is noticeably enhanced. Thus, the superior efficiency of saccharide-mediated cellular uptake offers another useful approach for delivery of therapeutic agents.

## 5. Cellular uptake through disulfide exchange on cell surface

Thiol groups exist in many proteins, are capable of folding and stabilizing protein’s structure by forming internal disulfide bonds. Particularly for proteins located on cell membrane, which have to face strong oxidants in the extracellular milieu, thiols play an important role in response to exogenous oxidative stress and make protein stability (Matthias & Hogg, 2003). Moreover, thiol groups have been reported to participate in the activation of intracellular signal pathways and alteration of protein function through disulfide exchange reactions at the plasma membrane (Filomeni et al., 2003; Hogg, 2003). The reversible nature of disulfide exchange reactions has been exploited in a number of ways for advanced drug delivery since Kichler et al. firstly reported the reactive cell surface thiol groups may aid absorptive endocytosis in 1995 (Kichler et al., 1995; Saito et al., 2003). Actually, Feener et al. has observed the phenomenon early in 1990 that cleavage of disulfide bonds in a disulfide-bridged macromolecule occurs at the plasma membrane prior to endocytosis, indicating that exofacial thiols may mediate a natural mechanism for cellular internalization of extracellular compounds (Figure 4) (Feener et al., 1990).

A variety of synthetic biomolecules, including small molecules (e.g. fluorescent dyes), macromolecules (e.g. peptides) and drug carriers (e.g. nanoparticles, polymers) that present thiol-reactive moieties, exhibited enhanced cellular association and internalization. For example, fluorescent probes (carboxyfluorescein) that cannot enter cells were equipped with cyclic disulfides, showed significant increase in uptake efficiency through a disulfide exchange with cellular external thiols (Gasparini et al., 2015). A protein kinase C peptide inhibitor that was not cell permeant displayed the capability of crossing cell membranes when an activated cysteine was introduced into its sequence (Aubry et al., 2009). The same result was observed for a longer peptide (up to 20 mer). The attachment of a single asparagusic acid (AspA) residue to BH3 domain peptides dramatically improves their transport across the cellular membrane. The mechanism studies revealed that cyclic disulfides are covalently bound to the TFR, subsequently, the cargoes were transported across the cellular membrane (Abegg et al., 2016). Torres et al. reported that the cellular uptake of peptide nucleic acid (PNA) anti-miRs, which were thought to be poorly taken up by cells in culture in the absence of transfection agents, can be dramatically increased in Huh7 cells and HEK293ET cells by simple addition a terminal-free thiol group (e.g. Cys residue) (Torres et al., 2012). Thiolated modification was also used to increase the delivery efficiency of chitosan nanoparticles. When *N*-acetylcysteine was covalently bound to chitosan to form chitosan thiomer polyplexes as a non-viral gene carrier, the transfection efficacy was raised 2.5-fold in comparison to polyplexes of unmodified chitosan (Loretz et al., 2007). Trimethyl chitosan nanoparticles with cysteine modification also showed higher permeation enhancing effects due to thiol-disulfide exchange reactions than that of non-thiolated counterparts (Yin et al., 2009). In addition, The presence of the thiols/disulfide bonds in poly(methacrylic acid) (PMA) capsules has been shown to enhance cellular contact by interacting with exofacial thiols, which may facilitate cell membrane wrapping, leading to internalization of the capsules (Yan et al., 2011).

Besides thiol groups, maleimide moiety was also thiol-reactive, which can form covalent bonds with cystines on surface of the cellular membrane. Fretz et al. reported that thiol-reactive small molecules, Alexa Fluor 488-C5-maleimide dye, can be conjugated to plasma membrane thiol groups in the absence of endocytosis at 4 °C (Fretz et al., 2007). Moreover, when liposomes were modified with a small amount of maleimide, the cellular uptake and drug-delivery efficiency were significantly enhanced (Li & Takeoka, 2014). The studies of the mechanism revealed that the interaction of maleimide with cellular thiols triggered alternative cellular internalization via thiol-mediated transport. The cell-surface thiols facilitated the cellular uptake of maleimide-modified liposomes.

Compared with number of cases which showed thiols enhanced cellular uptake *in vitro*, the *in vivo* studies of thiol-reactive biomolecules were very little. Even so, several examples have indicated the great potential for the application of thiol-reactive headgroups modified biomolecules *in vivo*. Reactive thiol groups have been immobilized on the polymeric structure to develop thiomers as a category of mucoadhesive polymers (Bernkop-Schnürch et al., 2004). They can tightly adhere to the intestinal mucus layer for a prolonged time, hence providing a steep drug concentration gradient at the absorption sites and exerting an additional permeation enhancing effect. Thiolated trimethyl chitosan nanoparticles have been used to deliver insulin via oral administration (Yin et al., 2009). Thiolated chitosan was also a promising tool for oral administration of P-gp substrates, the studies showed that the oral bioavailability of P-gp substrate Rho-123 was significantly increased through the thiolated chitosan delivery system (Föger et al., 2006). The disulfide-linked polymers of D-R9 have also shown the ability to enhance gene expression in mouse lung by intratracheal injection (Won et al., 2010). The high transfection efficiency and minimal toxicity *in vivo* suggested the potential *in vivo* applications as a gene carrier. In addition, the previous studies showed that, thiol-reactive maleimide headgroups, which were used in delivery of drug-carrier nanoparticles to therapeutic cells *in vivo*, eliciting enhancements in tumor elimination and increased the repopulation rate of hematopoietic stem cell grafts with very low doses of adjuvant drugs in mice (Stephan et al., 2010).

Although lots of questions still remain to be addressed, such as the detailed mechanism of thiol-mediated uptake (a new route or known endocytotic routes), it is clear that the promising field has given light to therapeutic biomolecule development because that thiol-reactive group's modification does enhance delivery efficiency through utilizing cell surface thiols and the effect does be widespread based on the strategy can be applied to a range of chemically distinct biomolecules and used to target numerous cell lines.

## 6. Conclusions and perspectives

We have shown various delivery strategies according to the interaction with different components of plasma membrane. However, what should be noted is that multiple components of plasma membrane may be involved in the cellular uptake of the same chemic entity during the progress of interaction. For instance, positive charged CPPs were adsorbed to negatively charged cell membrane before entering cells. The electronegativity of cell membrane mainly comes from sulfates and carboxylates of GAG, and phosphates in the glycerol backbone region of the lipid bilayer. Therefore, the uptake of CPPs-mediated may be related with both the glycerol backbone of lipid bilayer and GAG. Furthermore, the cargoes and the CPPs are often conjugated through a disulfide bridge, which could also absorb cargoes to cell membrane through the thiol/disulfide exchange. Thus, the uptake of disulfide-linked CPPs cargoes complexes may benefit from both anionic membrane and cell-surface thiols (Aubry et al., 2009). Jha et al. reported a cationic cysteine-rich CPP that exhibited higher cellular internalization than that of other well studied CPPs such as TAT, penetratin and octaarginines (Jha et al., 2011). Structure-activity relationship studies revealed that apart from positive charge in the peptide, cysteine residues play an important role in maintaining the carrier function. Moreover, siCPDs is a cell-penetrating poly-(disulfide) which was firstly developed by Bang et al. in 2013, and after that it has been used to deliver a variety of cargoes including small molecules, proteins and nanoparticles (Bang et al., 2013; Gasparini et al., 2014; Fu et al., 2015). Based on the fact that insensitivity to endocytosis inhibitors, Gasparini et al. proposed the thiol/counterion-mediated cellular uptake mechanism (Sakai et al., 2003; Gasparini et al., 2014). In other words, anionic membrane and exofacial thiols were involved in the cellular uptake of siCPDs delivery systems.

On the other hand, some ammonium groups are located on the exterior of plasma membrane, such as the primary amine in PS/PE that is another important component of polar headgroups in glycerophospholipids and sphingolipids of lipid bilayers, and some ammonium groups in Lys that is basic amino acid of trans-membrane proteins. To all of our best knowledge, there are no papers about utilizing amines on cell surface to enhance cellular uptake. However, Gao et al.’s publication about targeting bacteria via iminoboronate chemistry aroused our interest (Bandyopadhyay et al., 2015). In their studies, the 2-acetylphenylboronic acid (2-APBA) covalently linked to the primary amino group of PE to form an iminoboronate, therefore realized the selectively labeling of bacteria. Taking the presence of large nucleophilic primary amino groups on cell surface into consideration, it is expected that 2-APBA facilitate chemic entities enter cell. In our preliminary study (no published), 2-APBA was linked to FITC labeled BSA through a long fatty chain. The confocal laser scanning microscopy (CLSM) results showed that the modification of 2-APBA enables BSA to enter HeLa cell. On the contrary, BSA alone did not enter cells under experimental conditions. The preliminary results indicated that ammonium groups on cell surface can be used to facilitate cellular uptake. More research about the uptake of 2-APBA modified proteins is still underway.

Finally, it is worth noting that efficient membrane permeation is likely necessary for bioavailability that is the key factor for evaluating the developmental potential of drug (Veber et al., 2002). Here various drug delivery systems were classified according to the interaction with different components of plasma membrane, and some delivery strategies were presented that may have implications in the development of drug by improving drugs to pass through the membrane barrier. In summary, anionic cell membrane can be utilized to facilitate uptake of Arg-rich chemic entities by electronic interaction; pro-drug strategies can enhance cell uptake through increasing lipophilicity; proteins located on plasma membrane as transporters or receptors can improve cell uptake via ligand-receptor interaction; drugs or drug carriers modified with ligands targeting saccharides on cell membrane can enter cell by attraction with saccharides; chemic entities containing thiol-reactive moieties showed enhanced cellular uptake via disulfide exchange on cell surface. However, another important issue that is not discussed here is endosomal escape, which is perhaps another challenging barrier against the delivery of macromolecules or drug carriers. Although a great number of strategies have been developed to solve this problem, even several delivery strategies showed efficient in mice, there is still a long way to go before cellular uptake is mastered. On the other hand, medicine is currently entering the age of precision and personalized treatments; it is desirable to develop a novel delivery method based on the specific components of the diseased cell membrane, and thus to improve cellular uptake and achieve cell and tissue-selective targeting. In order to achieve this goal, additional research should be carried out to accurately understand the interactions of different chemical entities with cell membranes.

## Supplementary Material

Revised_SI.pdf
