# Supplementary material for: Improving cellular uptake of therapeutic entities through interaction with components of cell membrane
Source: Drug Deliv. 2019 Mar 24;26(1):328–42. doi: 10.1080/10717544.2019.1582730 (PMC6442206; doi:10.1080/10717544.2019.1582730)
Supplement: Revised_SI.pdf [file IDRD_A_1582730_SM7356.pdf]

Improving cellular uptake of therapeutic entities through interaction with components of cell membrane

Renshuai Zhang, *,† Xiaofei Qin, † Guojun Pan, *,‡ Fandong Kong, # Pengwei Chen #

† Key Laboratory of Flexible Electronics & Institute of Advanced Materials, Jiangsu National Synergetic Innovation Center for Advanced Materials (SICAM), Nanjing Tech University, Nanjing 211800, P.R. China

‡ School of Life Sciences, Taishan Medical University, Tai'an 271000, P.R. China

Key Laboratory of Biology and Genetic Resources of Tropical Crops, Ministry of Agriculture, Institute of Tropical Bioscience and Biotechnology, Chinese Academy of Tropical Agriculture Sciences, Haikou 571101, P.R. China

* Corresponding Author

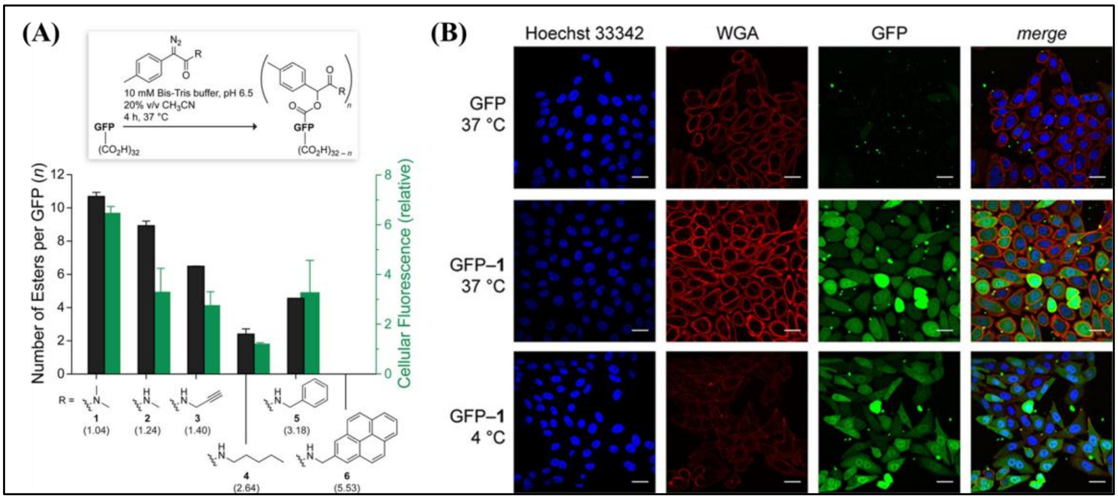

Figure S1. (A) Bar graph showing the extent of esterification of GFP with diazo compounds 1-6 (black) and the internalization of the ensuing esterified GFPs into CHO-K1 cells (green). (B) Images of the cellular internalization of GFP and its esterified variants. Reproduced with permission from ref (Mix *et al.*, 2017). Copyright 2017 American Chemical Society

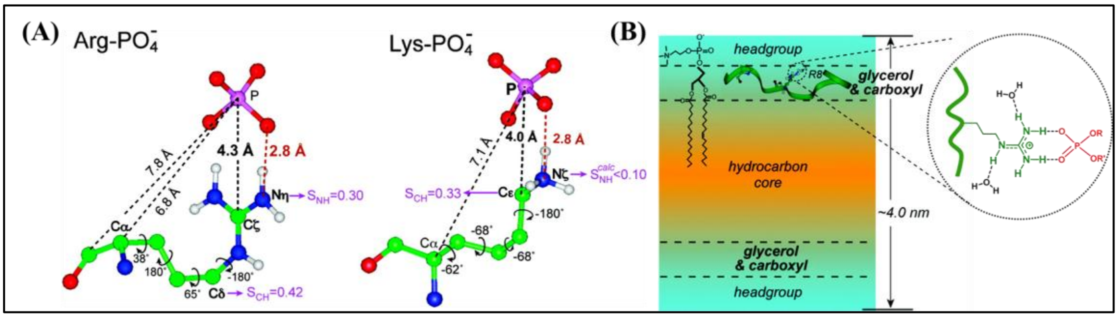

Figure S2. (A) Low-temperature side chain conformation and phosphate interaction of Arg10 and Lys13 in penetratin. (B) Model of TAT structure and dynamics in DMPC/DMPG bilayers. Reproduced with permission from ref (Su *et al.*, 2009, 2010). Copyright 2009, 2010 American Chemical Society.

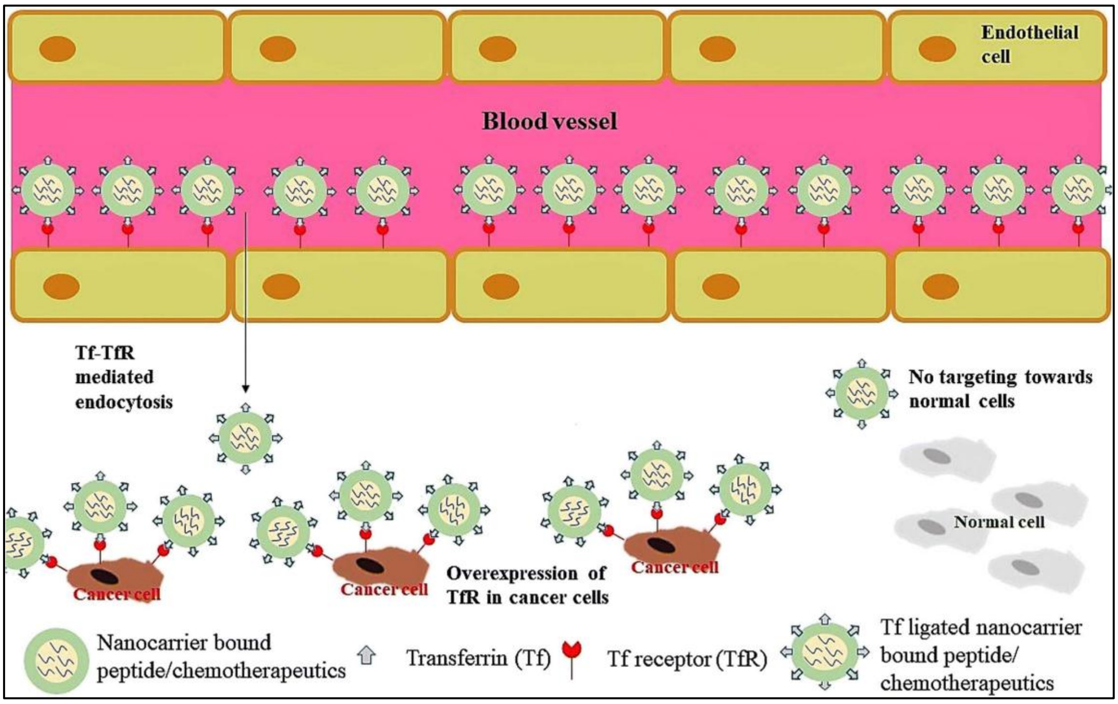

Figure S3. Improved uptake and accumulation of Tf-nanocarriers onto the TfR overexpressed tumor cell, whereas minimized targeting to normal cells. Reproduced with permission from ref (Choudhury *et al.*, 2018). Copyright 2018 Springer Nature.

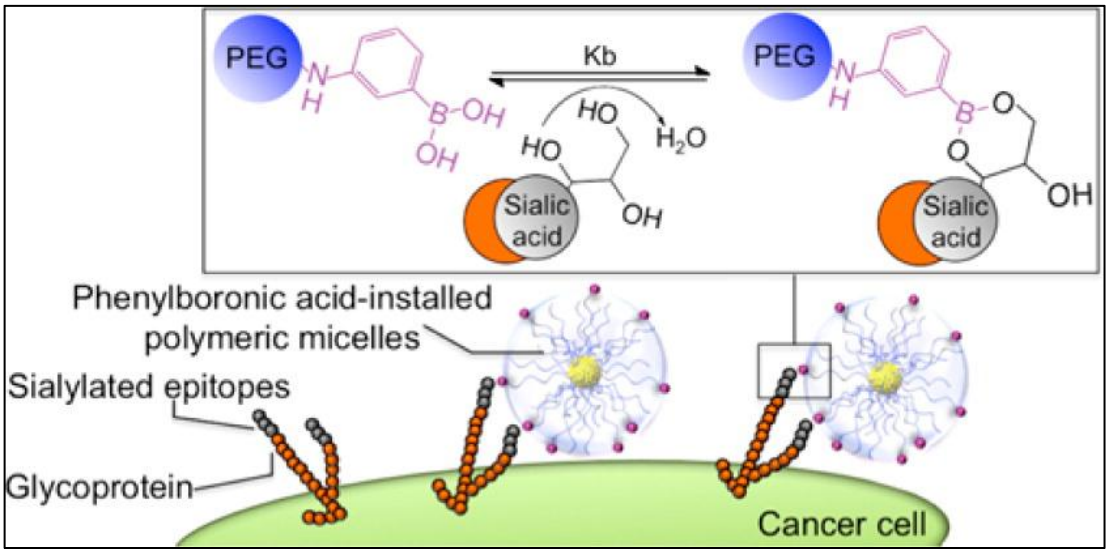

Figure S4. PBA-installed micellar nanocarriers for targeting sialylated epitopes overexpressed on cancer cells. Reproduced with permission from ref (Deshayes *et al.*, 2013). Copyright 2013 American Chemical Society.

Ref

Choudhury H, Pandey M, Chin PX, et al. (2018). Transferrin receptors-targeting nanocarriers for efficient targeted delivery and transcytosis of drugs into the brain tumors: a review of recent advancements and emerging trends. Drug Delivery and Translational Research 8**:**1545-1563.

Deshayes S, Cabral H, Ishii T, et al. (2013). Phenylboronic Acid-Installed Polymeric Micelles for Targeting Sialylated Epitopes in Solid Tumors. Journal of the American Chemical Society 135**:**15501-15507.

Mix KA, Lomax JE, Raines RT (2017). Cytosolic Delivery of Proteins by Bioreversible Esterification. Journal of the American Chemical Society 139**:**14396-14398.

Su Y, Doherty T, Waring AJ, et al. (2009). Roles of Arginine and Lysine Residues in the Translocation of a Cell-Penetrating Peptide from ^{13}C, ^{31}P, and ^{19}F Solid-State NMR. Biochemistry 48**:**4587-4595.

Su Y, Waring AJ, Ruchala P, et al. (2010). Membrane-Bound Dynamic Structure of an Arginine-Rich Cell-Penetrating Peptide, the Protein Transduction Domain of HIV TAT, from Solid-State NMR. Biochemistry 49**:**6009-6020.
